# Electron Tomography of Pencil-Shaped GaN/(In,Ga)N Core-Shell Nanowires

**DOI:** 10.1186/s11671-019-3072-1

**Published:** 2019-07-12

**Authors:** Lars Nicolai, Žarko Gačević, Enrique Calleja, Achim Trampert

**Affiliations:** 1 Paul-Drude-Institut für Festkörperelektronik, Leibniz-Institut im Forschungsverbund Berlin e.V., Hausvogteiplatz 5-7, 10117 Berlin, Germany; 2 ISOM-ETSIT, Universidad Politécnica de Madrid, Avda. Complutense s/n, 28040 Madrid, Spain

**Keywords:** Electron tomography, STEM, (In,Ga)N/GaN nanowire, Dot-in-a-wire, Morphology

## Abstract

The three-dimensional structure of GaN/(In,Ga)N core-shell nanowires with multi-faceted pencil-shaped apex is analyzed by electron tomography using high-angle annular dark-field mode in a scanning transmission electron microscope. Selective area growth on GaN-on-sapphire templates using a patterned mask is performed by molecular beam epitaxy to obtain ordered arrays of uniform nanowires. Our results of the tomographic reconstruction allow the detailed determination of the complex morphology of the inner (In,Ga)N multi-faceted shell structure and its deviation from the perfect hexagonal symmetry. The tomogram unambiguously identifies a dot-in-a-wire configuration at the nanowire apex including the exact shape and size, as well as the spatial distribution of its chemical composition.

**Electronic supplementary material:**

The online version of this article (10.1186/s11671-019-3072-1) contains supplementary material, which is available to authorized users.

## Introduction

The ongoing process of miniaturization of optoelectronic devices has given rise to the development of complex, three-dimensional (3D) nanostructures. In this respect, nanowires (NWs) are promising candidates to realize high-quality quantum well or quantum dot (QD) structures due to their large surface-to-volume ratio associated with an efficient strain relaxation in axial or radial NW heterostructures [1–3]. Recent improvements in selective area growth (SAG) by molecular beam epitaxy (MBE) on GaN-on-sapphire templates has led to the fabrication of ordered and uniform GaN NW arrays with either flat- or pencil-shaped top [4, 5]. The latter has been used to fabricate (In,Ga)N/GaN shell structures grown on the multi-faceted tip of the GaN core providing an alternative solution for the growth of QDs. Taking advantage of the reduced NW diameter and the corresponding possibility to grow heterostructures with short sections of low-band gap (In,Ga)N material inserted in GaN barriers leads to the formation of so-called dot-in-a-wire (DIW) structures. Depending on the actual dimensions, this DIW configuration enabled the emission of linearly polarized single photons by using the major benefit to easily probe only one single NW instead of an NW ensemble [6–8]. A detailed microstructural analysis of these DIW heterostructures is nevertheless necessary to understand the influence of the NW morphology, the shell thicknesses, and the local chemical composition on the single photon emission characteristics.

The transmission electron microscope (TEM) is a frequently used and powerful tool to obtain information about the structure and chemical composition of such nanostructures on the atomic scale [9]. However, the lower symmetry of these 3D nanostructures compared to, for example, planar systems makes the interpretation of TEM micrographs far more difficult. A main characteristic is the transmission of the sample by the electron beam so that the structural information is projected into a two-dimensional image. Variations of the sample structure in the direction of the electron beam and in the order of the sample thickness, or lower, are very difficult or even impossible to detect directly. Electron tomography can circumvent this problem. Instead of using a single projection of the sample, a series of projections with different tilt angles to the object is recorded to reconstruct the 3D information of the sample. This enables new and advanced possibilities to describe and analyze the morphology and chemical composition of complex structures like core-shell NWs. So far, only a few publications have been published concerning electron tomography on NW structures [9–15] or embedded QD structures [16].

This work describes the application of electron tomography for the structural characterization of ordered GaN NWs containing an embedded (In,Ga)N shell. The sample preparation is explained herein in detail due to the challenge to make single NWs accessible for electron tomography by isolation without introducing damage. The surface morphology and crystal faceting of the NW are studied through the analysis of 3D surface representations of the outer GaN shell. The inner structure of the NW, i.e., the morphology of the (In,Ga)N shell as well as the spatial distribution of the chemical composition, is discussed with the help of two-dimensional slices of the reconstructed volume and complementary energy-dispersive x-ray (EDX) spectroscopy measurements.

## Methods

### Material

**Fig. 1:**
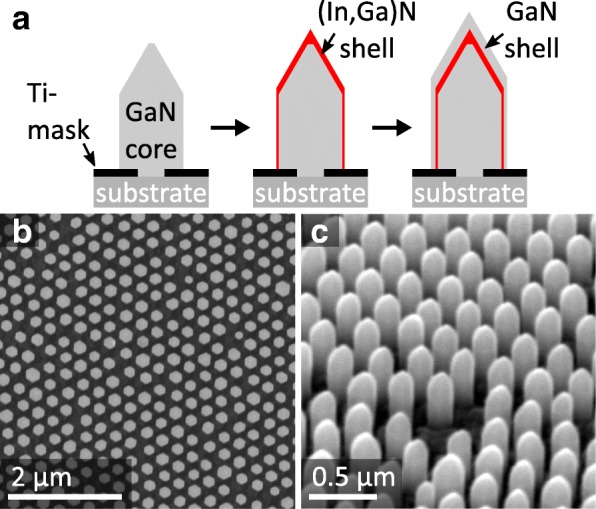
**a** Schematic of the NW growing process. SEM micrographs of **b** an array of GaN/(In,Ga)N NWs from top view and **c** 45° tilted view with higher magnification

The GaN/(In,Ga)N NWs were grown on a commercial GaN-on-sapphire (0001) template (LUMILOG) with a GaN buffer layer thickness of 3.3 μm by plasma-assisted molecular beam epitaxy (PAMBE). In a first step, colloidal lithography was used to create a Ti nanohole mask building a hexagonal pattern. The subsequent SAG produced a periodic array of GaN NWs with pencil-like apex. The GaN NW cores were overgrown by a thin (In,Ga)N layer at lower growth temperature and then completed by a thin GaN capping layer without changing the temperature. A schematic of the growing process is shown in Fig. 1a. Details about the substrate patterning procedure and the SAG MBE process can be found elsewhere [6, 7, 17]. Figure 1 depicts two scanning electron microscopy (SEM) images of the sample showing the hexagonal array of NWs from top view (b) and with a 45° tilted view (c) in higher magnification. The SEM micrographs reveal a relatively homogeneous arrangement with only slight variations in shape and length. The average diameter of the NWs is about 180 nm, and the average height is about 500 nm.

### Tomography Needle Preparation

A sophisticated preparation technique is necessary to obtain the needle-shaped tomography specimen containing only one single NW. A round-shaped needle allows the maximum tilt range of 180° whereby the sample thickness is almost constant for all tilt angles. The focused gallium-ion beam (FIB) microscope enables this site-specific sample preparation. A dual-beam microscope system (JEOL JIB-4501) was employed for this work. The following preparation steps are based on the standard FIB lift-out technique with a subsequent thinning to obtain the needle-shaped specimen [18–20].

**Fig. 2:**
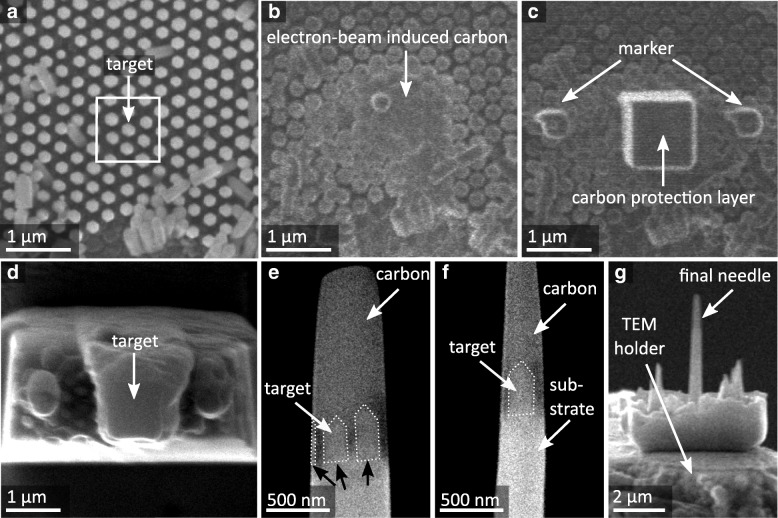
SEM micrographs showing **a** an array of NW and the selected area for the FIB needle preparation (white box), **b** the NW embedded in electron beam-induced carbon, **c** the gallium beam-induced carbon as thick deposition layer on top of the selected area and two additional markers, **d** the isolated needle ready for lift-out, **e**, **f** the thinned tomography needle (black arrows indicate NW positions), and **g** an overview of the final tomography needle

The various preparation steps are summarized in Fig. 2. Initially, the area of interest is selected, which is identified by an undisturbed and almost perfect hexagonal arrangement of a few NWs (marked with a white box in Fig. 2a). This selected probe volume must be filled with carbon to protect the NW during the FIB milling process [21]. Carbon depositions were carried out in two steps in order to reduce the Ga contamination: (i) firstly, by electron beam-induced carbon deposition to fill the volume between the NWs (Fig. 2b), and (ii) subsequently, with carbon deposition induced by gallium beam to create a ca. 1-μm-thick protective layer on top of the selected area (Fig. 2c). Additional carbon markers were produced to simplify the orientation during the following preparation steps.

After carbon deposition, the FIB milling was introduced to isolate the protected area (Fig. 2d) and to fabricate a rectangular-shaped needle that contains several NWs. A micro-manipulator (Kleindiek Nanotechnik GmbH) was employed to transfer the needle to the tomography holder. Thereby, the needle axis must be carefully adjusted parallel to the goniometer rotation axis so that the same focus is applicable for a large sample area. Additionally, the NW [0001] *c*-axis is perpendicular to the substrate and therefore parallel to the needle axis. This relationship was used to determine the crystal directions with respect to the tomogram axis. Posterior measured selected area diffraction (SAD) patterns showed a tilt of the NW [0001] *c*-axis to the rotation axis of the tomogram of only 2.2°.

Further thinning steps were carried out to isolate one single NW, to round off the needle, and to obtain eventually electron transparency (cf. Fig. 2e, f). Figure 2g shows the final tomography needle.

### Electron Tomography

Tomographic acquisition and microstructural analysis were performed with a TEM (JEOL JEM-2100F) operating at 200 kV. The microscope is equipped with a scanning unit including a bright-field (BF) and high-angle annular dark-field (HAADF) detector as well as a 50-mm^2^ X-ray detector (JEOL EX-24065) for EDX spectroscopy. The HAADF scanning transmission electron microscopy (STEM) mode is chosen due to the predominantly chemical contrast [22]. The monotonic relationship of intensity to mass density and thickness of the object is a prerequisite for electron tomography and is known as “projection requirement” [23].

A series of 89 HAADF micrographs was recorded with steps of 2° between each measurement. This tilt series that covers the full range of 180° is made possible by a special tomography holder (Model 2050 from E.A. Fischione Instruments Inc.) supporting the prepared geometry of the sample needle. Each STEM micrograph is captured with a 2048 × 2048 pixel resolution; a pixel dwell time of 30 μs, i.e., a full scanning time of 127 s per image; a spot size of 0.5 nm; and an electron acceptance angle of 70 to 180 mrad according to the manufacturer’s manual. The micrographs were binned (4 × 4 binning = 512 × 512 final resolution) to improve the signal-to-noise ratio as well as the computing speed of the 3D reconstruction. All micrographs are manually aligned to each other so that the needle axis is selected as the rotation axis for the Radon transformation. The tomogram is calculated and visualized by a tomography software package (IMOD) [24]. An advanced rendering of 3D structures is performed with the free and open-source computer graphics software Blender (Blender Foundation).

Within this work, two different methods are applied for visualization. Two-dimensional slices are extracted from the 3D reconstructed volume. Such slices have a final thickness over which the voxel (3D pixel) intensities are integrated to improve the signal-to-noise ratio. The ideal slice width is a compromise between noise reduction and contrast blurring due to an averaging of sample variations perpendicular to the slice. Another visualization method is the isosurface representation. It is utilized in case of sufficient contrast between two adjacent materials. In general, the isosurface is a 3D surface representation of voxels with constant intensity. The intermediate intensity between two materials is chosen in order to construct an isosurface reproducing the interface of the adjacent materials.

Although HAADF micrographs are used as basis of the tomogram calculation, the reconstructed intensity distribution originates not only from the chemical composition of the sample. Crystalline defects in the sample [25] or, on the other hand, a misalignment of the micrographs and distortions of the micrographs due to sample drift or magnetic field disturbances influence the reconstructed intensities and thus the final resolution. The same applies to intrinsic reconstruction errors like cupping artifacts [26] or the limit of spatial resolution of the tomogram due to the Crowther criterion [27] (limited sampling). The latter should be considered especially for thick tomography needles of several hundreds of nanometers. If the size of the object to be reconstructed is increased, the tomogram resolution becomes worse if the number of micrographs is fixed.

## Results and Discussion

### Surface Morphology and Crystal Faceting

**Fig. 3:**
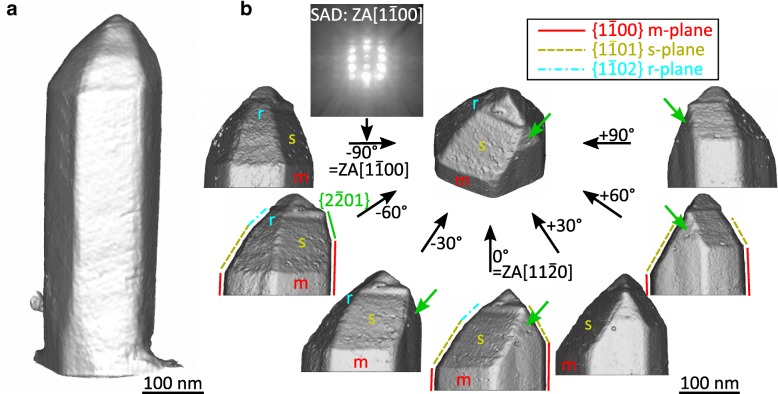
Isosurface representation of **a** a single NW and **b** a NW apex with a perspective view in the center and various viewing angles along low index directions of GaN (ZA, zone axis). Additionally, some exemplary *m*-, *s*-, and *r*-plane facets are labeled (green arrows indicate a facet of $\left\{2\overline{2}01\right\}$ -type)

Figure 3 a and b show the isosurface representations of a complete NW and of a NW apex in perspective view (center) and in various views along low index directions in steps of 30°. The figure reveals the outer crystal shape and surface faceting, respectively. The lower section of the reconstruction displays the expected hexagonal cylinder of the NW, with regular non-polar $\left\{1\overline{1}00\right\}$ *m*-plane surface facets. The crystal facets and planes are determined on the basis of the corresponding electron diffraction pattern taken simultaneously with HAADF images. An example of such an SAD pattern is given for the − 90° orientation, i.e., along the [$1\overline{1}00\Big]$ zone axis (cf. Fig. 3b). The NW top reflects a pyramidal shape consisting of $\left\{1\overline{1}01\right\}$ *s*-plane and $\left\{1\overline{1}02\right\}$ *r*-plane facets, which are however not perfectly symmetrically positioned to each other. A very small, triangular-shaped surface facet (marked by green arrows in Fig. 3b) is situated close to the NW tip which most likely represents a $\left\{2\overline{2}01\right\}$-type facet. Such slight asymmetries in the pyramidal tip shape are frequently found on the sample (cf. Fig. 1). The reason for this deviation is associated with defect interactions as discussed in the next paragraph.

Above the labeled *r*-plane facet and the $\left\{2\overline{2}01\right\}$ facet, an irregular NW “hat” is formed at the very top. High-resolution (HR) TEM measurements on a lamella TEM sample containing several NWs of the same wafer demonstrate the presence of stacking faults and the change of the crystal lattice from hexagonal to cubic at the NW top region (not shown here). These structural changes are in accordance with our previous observations which are explained by the instability of the crystal phase due to the significantly lower growth temperature used for the growth of the GaN outer shell (ca. 625 °C) compared to the GaN core (ca. 850 °C) [5, 7].

### Internal (In,Ga)N Shell Structure

The tomogram of the NW has been used to extract information about the internal structure of the (In,Ga)N shell, its chemical composition, and spatial distribution. A 3D isosurface representation of the shell structure cannot be easily accessed due to the low voxel contrast between (In,Ga)N shell and GaN matrix material. Therefore, as an alternative, the internal shell structure is visualized by extracting thin slices cut out of the reconstructed 3D tomogram.

**Fig. 4:**
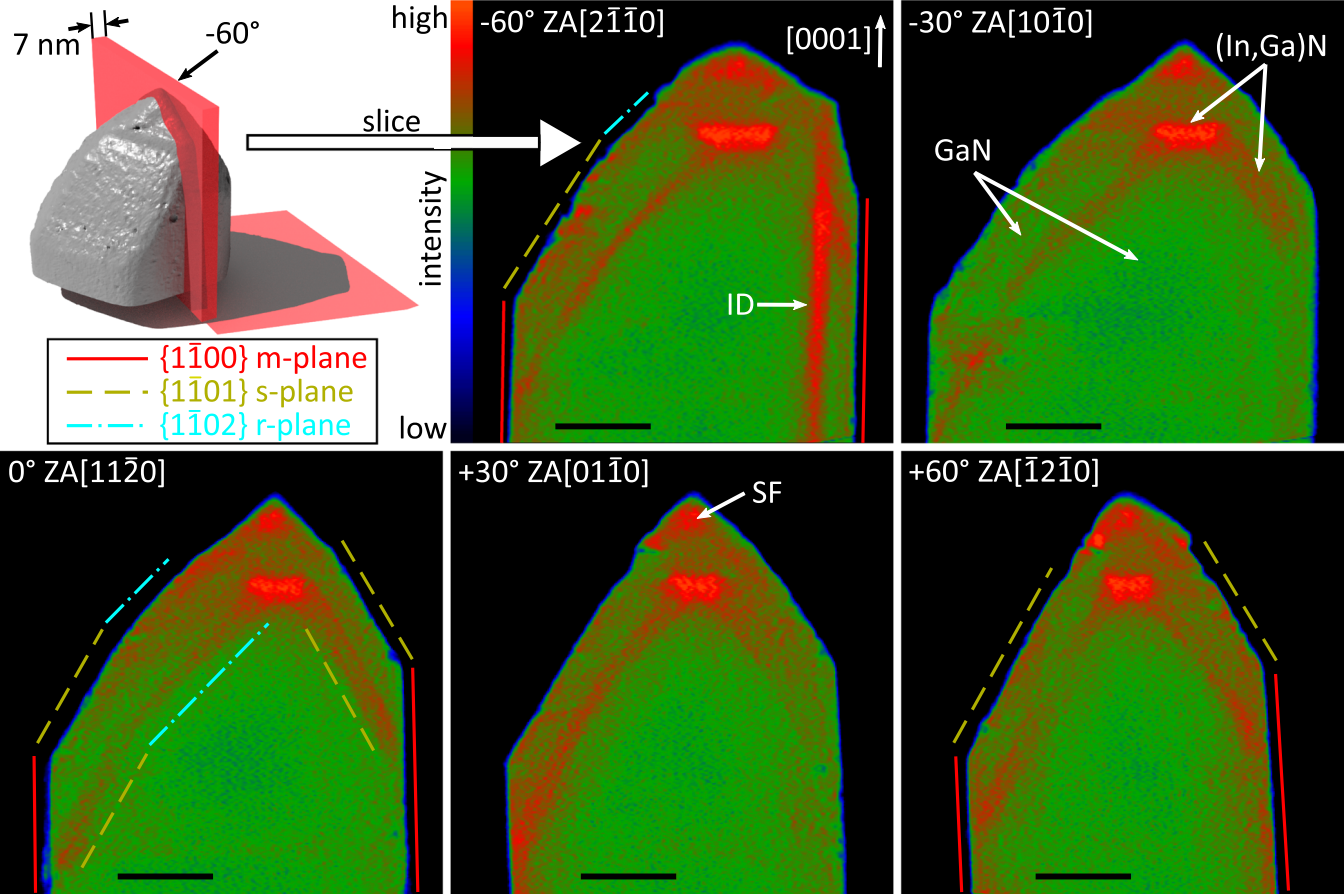
Cross-sectional slices through the tomogram. A 3D rendered representation of the NW and a slice (upper left corner) specifies the spatial position of the − 60°-tilted slice. All slices are rotated around an axis which pierces through the NW tip and which is parallel to the [0001] NW growth axis. An inversion domain (ID) and the location of stacking faults (SF) are labeled. The slice orientations correspond to the labeling of Fig. 3. The length of the black scale bar corresponds to 50 nm

Figure 4 shows as an example of five cross-sectional slices through the NW tip and along the [0001] wire axis. Each slice has a thickness of about 7 nm. The orientation of the slices has been selected in a way that the hexagonal sixfold symmetry is taken into account. Hence, the slices are rotated by 30° to each other—in correspondence to the labeling introduced in Fig. 3b. To illustrate this point further, a 3D rendered image of the NW together with the spatial position of the slice that is − 60° tilted (i.e., the slice parallel to the $\left(\overline{2}110\right)$ lattice plane) is additionally given in the figure.

The reconstructed voxels of (In,Ga)N have slightly higher intensities compared to those of GaN. Consequently, referring to the color code of Fig. 4, GaN is presented in green whereas In-containing layers appear reddish for clarification. The cross-sectional slices demonstrate the core-shell structure of the NW. Due to the reduction in growth temperature for (In,Ga)N overgrowth, it is reasonable to assume that the morphology of the GaN core remains unchanged and the (In,Ga)N growth proceeds in a conformal manner. Thus, the (In,Ga)N inner shell as well as the GaN outer shell roughly replicates the morphology of the GaN NW core. Specifically, the (In,Ga)N layer forms a complete *m-*plane shell around the wire turning into *s*- and *r*-plane facetted pyramidal shells at the tip of the NW. The tip of the inner shell is expanded forming a so-called (In,Ga)N DIW configuration with the shape of an inverse truncated pyramid with hexagonal base composed of *c*-plane facets as upper and lower boundaries (see next paragraph).

Moreover, Fig. 4 gives an overview of the various (In,Ga)N layer thicknesses. The *m*-plane shell is only 1 nm thick (in accordance with HAADF STEM micrographs along the $\left\langle 11\overline{2}0\right\rangle$ direction, cf. Additional file 1: Figure S1) whereas the *s*- and *r*-plane facets have thicknesses ranging from 8 to 14 nm. This thickness difference is a consequence of heterogeneous growth rates [28, 29] of the different facets and the shadowing effect induced by the low indium diffusion during MBE growth [30]. Furthermore, the indium atoms are not homogeneously distributed along the shell structure, because the indium incorporation rate depends on the facet orientation with the highest value in *c*-plane layers [31]. Additionally, it seems that in some areas of the shell, the concentration is higher close to the interfaces. It should be mentioned that the *m*-plane shell is only poorly resolved in the reconstruction. The rotation axis of the Radon transformation was chosen to penetrate the NW tip to achieve the best tomography resolution in the center of the NW according to the Crowther criterion.

The − 60°-oriented slice shows a strip of high intensity with a width of 10 nm. This strip was also visible as bright contrast in the HAADF images of the tilt series. Dark-field g_0002_ measurements indicate the presence of inversion domain boundaries which is in accordance with the observation of similar structures by Kong *et al.* [32]. It has been found that the inversion domain was induced by an unintended atomic layer of titanium (mask residues) being located between substrate and NW. The electron tomography of this inversion domain reveals the shape of an elliptical cylinder as it will be demonstrated in the following.

**Fig. 5:**
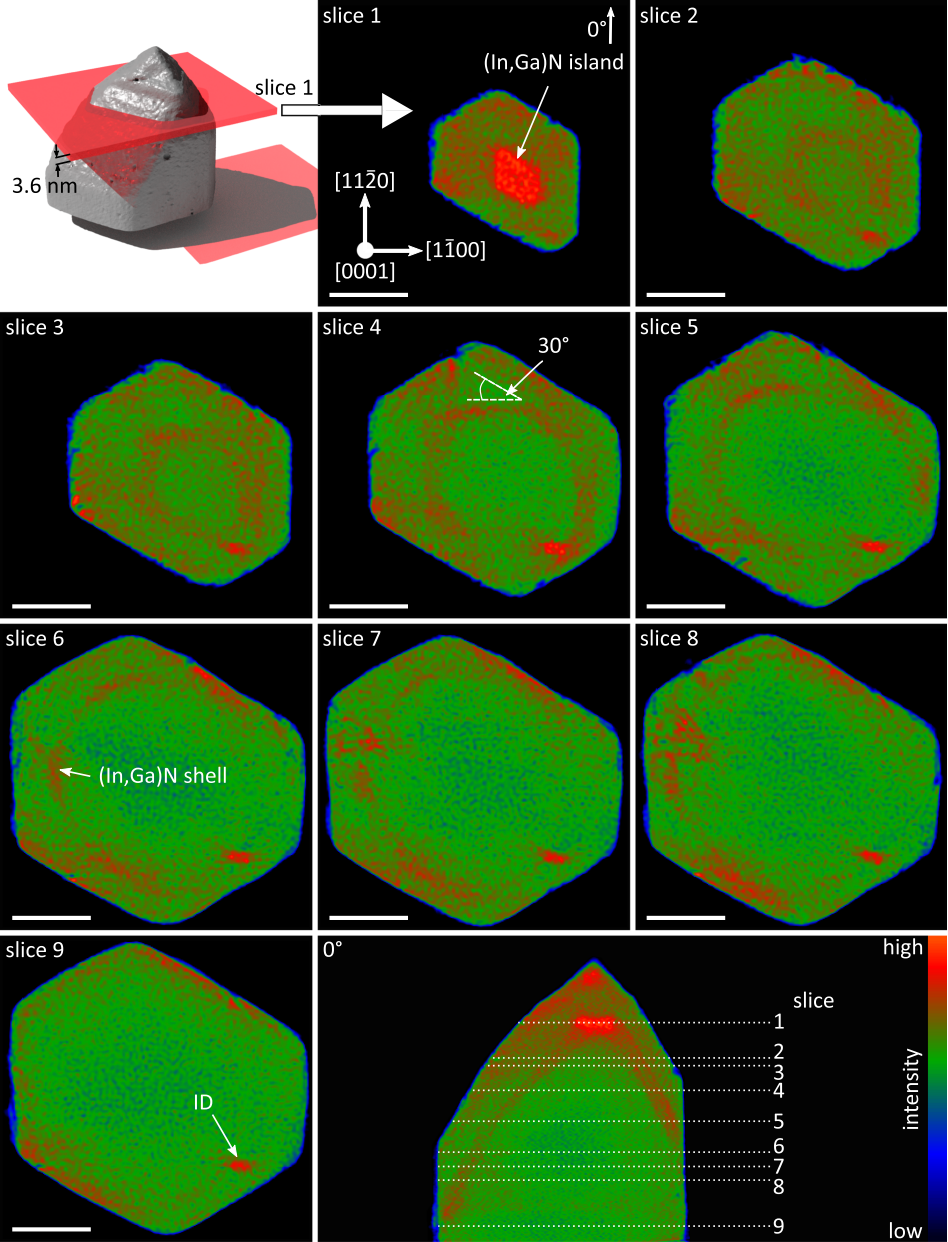
Plan-view slices through the tomogram. A 3D rendered representation of the NW and a slice (upper left corner) specify the spatial position of slice 1. All slices are perpendicular to the [0001] direction, and the different slice positions are labeled in the cross-sectional slice (bottom right). The slices have a width of 3.6 nm. The length of the white scale bar corresponds to 50 nm

A series of plan-view slices through the tomogram perpendicular to the NW axis were made in addition to the cross-sectional sections in order to obtain a full 3D imagination of the shell structure. Nine slices at different heights are shown in Fig. 5 together with a depiction of the spatial position of the first slice, together with a cross-section representing the different height positions. All plan-view slices have a width of 3.6 nm.

The consideration of Fig. 5 offers two new insights into the inner NW structure which was not experimentally accessible without electron tomography. First, it is directly evident that the NW diameter and thus the plan-view slice area decreases from bottom to top, which is the result of the pencil-like shape of the NW. However, it is noteworthy that the sidewall close to the elliptical cylinder-like inversion domain stays at its position and changes its dimension slower than the other sidewalls. A comparison with the isosurface representation (cf. Fig. 3) shows that this sidewall corresponds to the outer GaN shell with the very elongated *m*-plane facet which turns into a triangular-shaped, $\left\{2\overline{2}01\right\}$-like facet (green arrow in Fig. 3). Therefore, it can be concluded that the presence of the inversion domain affects the overall growth kinetics resulting in a pinning of the closest located sidewall. Consequently, the center of the NW tip is shifted toward the inversion domain, and the opposite facets must turn at lower heights from *m*-plane to *s*- and *r*-plane facets to form the displaced NW tip.

Second, the (In,Ga)N shell is not always parallel to the *m*-, *r*-, or *s*-plane faceted GaN sidewall. In the lower part of the NW, the (In,Ga)N shell reproduces one-to-one the shape of the GaN core with *m*-plane facets just like the GaN outer shell. On the other hand, at the pyramidal tip of the NW, the inner (In,Ga)N shell deviates from the hexagonal shape of the GaN outer shell. For example, slice 4 in Fig. 5 shows that the GaN outer facet and the (In,Ga)N shell have facets which are 30° rotated to the expected orientation based on symmetry reasons. These facets correspond to semi-polar $\left\{11\overline{2}l\right\}$ facets. Regarding slice 1 and 2, the (In,Ga)N shell returns to a hexagonal shape toward the tip with two of the six facets only slightly pronounced. This deviation from the hexagonal shape is unexpected and can only be revealed by electron tomography. It is remarkable that the GaN outer shell does not fully replicate the shape of the inner (In,Ga)N shell, instead the NW shape changes to the expected hexagonal symmetry of a GaN NW.

### Dot-in-a-Wire Structure

**Fig. 6:**
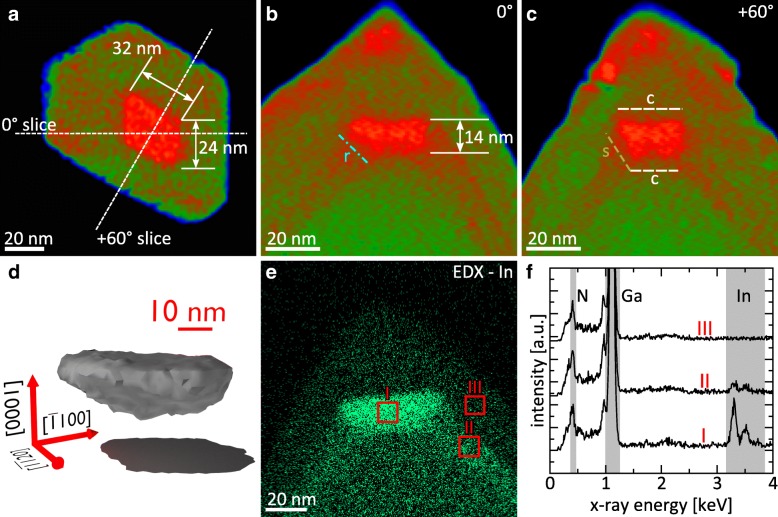
Magnified **a** plan-view and **b**, **c** cross-sectional tomographic slices of Figs. 4 and 5 revealing the morphology of the dot-in-a-wire structure. **d** 3D isosurface representation of the (In,Ga)N dot. The EDX measurements at a similar NW tip are presented in form of **e** an EDX map showing the spatial indium distribution and **f** an EDX spectra extracted from the map at three different regions: (I) in the (In,Ga)N dot, (II) in the (In,Ga)N shell, and (III) in the GaN outer shell

As previously shown in Figs. 4 and 5, an insertion of increased indium content is located at the tip of the (In,Ga)N shell. A more detailed view on this DIW structure is depicted in Fig. 6. The figure presents magnified versions of both the plan-view and the cross-sectional slices of the tomographic reconstruction. Additionally, it shows an isosurface representation of the 3D shape of the dot as well as the indium distribution measured by EDX. The EDX analysis is performed from the apex of a similar NW of the same wafer.

The three slices in Fig. 6a–c reveal the shape and dimensions of the dot. Based on the plan-view slice in (a), the dot almost displays the geometry of a parallelogram instead of a hexagon with two less pronounced sidewalls. The lengths of the two larger pairs of sidewalls are 32 nm and 24 nm, respectively. The height of the dot—as given by the two cross-sectional slices in (b) and (c)—is about 14 nm. In addition, the cross-sectional slices reveal a lateral broadening of the dot toward the top that is accompanied by a formation of *r*- and *s*-plane side facets whereby the bottom and top form *c*-plane facets. Thereby, the dot structure resembles an inverse truncated pyramid with a distorted hexagonal base. This 3D form of the nanodot is further illustrated by the isosurface representation in Fig. 6d, which confirms the faceted shape of the dot and additionally demonstrates that the lower *c*-plane facet exhibits a higher roughness.

Figure 6e and f reveal the result of the EDX measurement by means of an indium map in combination with spectra taken from positions inside the (In,Ga)N dot (I) and shell (II), as well as in the GaN outer shell (III). No intensity of the In-*Lα*_*1*_ line is detected in the GaN region (III). On the other hand, there is a tremendous difference in line intensity between shell and dot verifying the huge difference in indium concentration [7]. The indium content of the dot is roughly estimated at (24 ± 6)% (see Additional file 1: Figure S2 for more details). The EDX map therefore allows a clear spatial separation between the (In,Ga)N-shell and dot proving at the same time its faceted shape. Furthermore, the EDX map demonstrates that the voxels of high intensity in the tomogram very close to the NW tip do not arise from indium incorporation. This increase in intensity can be attributed to HAADF contrast arising from stacking faults at the disturbed “hat” region [25]. In addition, the tip has a much smaller thickness compared to the other parts of the NW leading to an overvaluation of the mass density of the tip region [26].

The 3D isosurface representation of the (In,Ga)N dot reveals a significant chemical roughness of the lower interface compared to the smooth *c*-plane interface on top (cf. Fig. 6d). The origin of this roughness can be linked to the nucleation mechanism of (In,Ga)N on the multi-faceted apex of the GaN NW core. While the (In,Ga)N growth on *m*-, *r*-, and *s*-planes occurs in a 2D mode due to the small indium concentration, the much higher indium content (In,Ga)N on *c*-plane results in the growth of strained 3D nuclei. These nuclei generate stress on its surrounding that will deform the interface and finally lead to the measured roughness.

## Conclusions

An (In,Ga)N/GaN core-shell NW has been investigated by electron tomography. The isosurface representation as well as tomographic slices allowed the determination of the faceting of the GaN outer shell and the (In,Ga)N inner shell. It has been shown that the symmetry of the NW is disturbed by the presence of a cylindrical inversion domain. Especially, a deviation of the expected hexagonal symmetry of the inner (In,Ga)N shell was clarified, which could only be resolved by electron tomography. In addition, differences of the (In,Ga)N shell thickness and the indium incorporation of the various facets were analyzed. Furthermore, the morphology of the (In,Ga)N DIW structure was characterized in detail. It has been found that the dot is faceted and contains a significantly higher indium content compared to the shell. A complementary EDX map was used to confirm the tomogram voxel intensities, which were influenced by, for example, stacking faults forming at the very NW tip that may be due to the low-temperature growth of the GaN outer shell.

The tomography analysis provides a complete figure of the complex core-shell structure of the investigated NW. The GaN core has a hexagonal shape including a pyramidal tip with slight deviations due to the influence of an inversion domain, and the (In,Ga)N shell replicates one-to-one the shape of the core. Unexpectedly, the GaN outer shell does not reproduce the shape of the inner (In,Ga)N shell and GaN core; instead, it is transformed into the expected shape based on hexagonal symmetry. The results demonstrate that electron tomography allows insights into the evolution of the core-shell structure formation during growth.

Future investigations in this field point at the problem of alloy stability and potential alloy fluctuations on the nanometer scale and their spatial distribution because they strongly affect the emission characteristics and optical properties. Therefore, our attempt could be to improve the spatial resolution and chemical sensitivity of our 3D reconstruction of the (In,Ga)N DIW structure to be able to detect indium alloy inhomogeneities and nanoclusters.

## Additional file

Additional file 1: **Figure S1.** Exemplary high-angle annular dark-field micrograph taken from the tomography tilt-series. The contrast of the micrograph was varied in a way that (A) the internal shell structure is visible showing that the GaN core is fully surrounded by an (In,Ga)N shell and (B) the carbon protection layer is visible. **Figure S2.** Energy-dispersive x-ray (EDX) analysis of the nanowire apex. The EDX maps of Ga and In were used to extract a line profile for both elements (left graph). The Ga signal without the DIW area was interpolated by a polynomial fit. Then, the Ga signal was normalized by dividing the Ga profile by the fit function. As a result, the right graph shows the reduction of the Ga signal within the DIW area by (12±3)%. Assuming a cylindrical shape of the DIW and the NW, the size of the DIW is about half the size of the total NW thickness at this height. Consequently, the indium content within the DIW can be roughly estimated to be (24±6)% assuming an exact stoichiometric composition. (PDF 259 kb)

## Data Availability

The datasets used and analyzed during the current study are available from the corresponding author on reasonable request.

## Abbreviations

(S)TEM: (Scanning) transmission electron microscope
BF: Bright-field
DIW: Dot-in-a-wire
EDX: Energy-dispersive x-ray
FIB: Focused ion beam
HAADF: High-angle annular dark-field
ID: Inversion domain
MBE: Molecular beam epitaxy
NW: Nanowire
PAMBE: Plasma-assisted MBE
QD: Quantum dot
SAD: Selected area diffraction
SAG: Selective area growth
SEM: Scanning electron microscopy
SF: Stacking faults
ZA: Zone axis
