# Supplementary material for: Electron Tomography of Pencil-Shaped GaN/(In,Ga)N Core-Shell Nanowires
Source: Nanoscale Res Lett. 2019 Jul 12;14:232. doi: 10.1186/s11671-019-3072-1 (PMC6626086; doi:10.1186/s11671-019-3072-1)
Supplement: Supplementary file 1 — Figure S1. Exemplary high-angle annular dark-field micrograph taken from the tomography tilt-series. The contrast of the micrograph was varied in a way that (A) the internal shell structure is visible showing that the GaN core is fully surrounded by an (In,Ga)N shell and (B) the carbon protection layer is visible. Figure S2. Energy-dispersive x-ray (EDX) analysis of the nanowire apex. The EDX maps of Ga and In were used to extract a line profile for both elements (left graph). The Ga signal without the DIW area was interpolated by a polynomial fit. Then, the Ga signal was normalized by dividing the Ga profile by the fit function. As a result, the right graph shows the reduction of the Ga signal within the DIW area by (12±3)%. Assuming a cylindrical shape of the DIW and the NW, the size of the DIW is about half the size of the total NW thickness at this height. Consequently, the indium content within the DIW can be roughly estimated to be (24±6)% assuming an exact stoichiometric composition. (PDF 259 kb) [file 11671_2019_3072_MOESM1_ESM.pdf]

Additional File 1: Supplementary Material

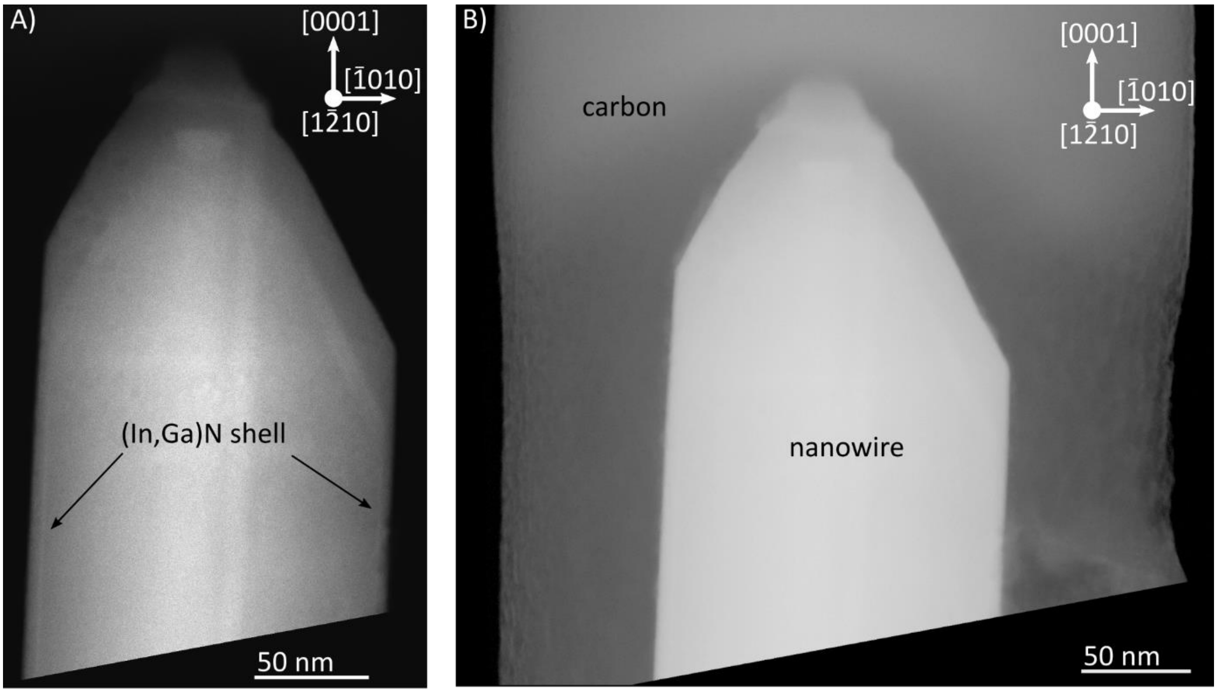

Figure S1: Exemplary high-angle annular dark-field micrograph taken from the tomography tilt-series. The contrast of the micrograph was varied in a way that (A) the internal shell structure is visible showing that the GaN core is fully surrounded by an (In,Ga)N shell and (B) the carbon protection layer is visible.

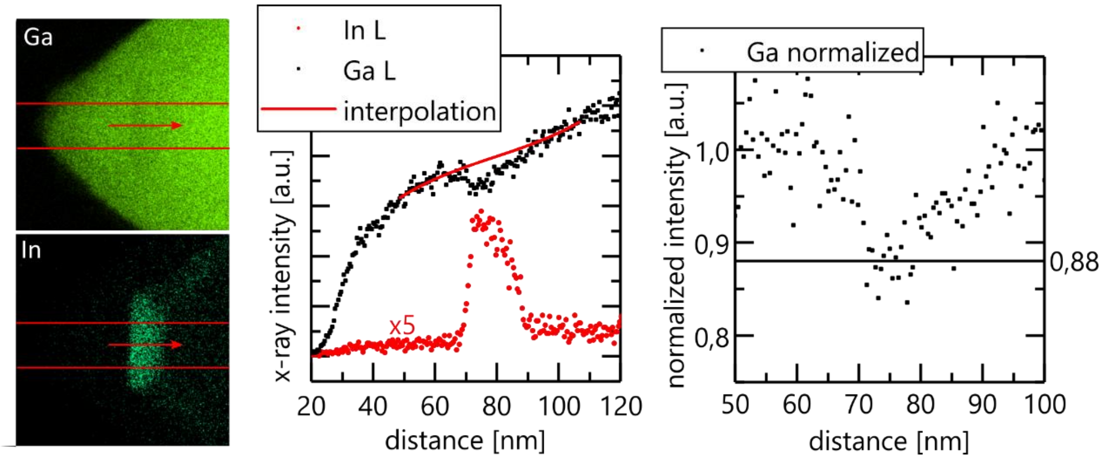

Figure S2: Energy-dispersive x-ray (EDX) analysis of the nanowire apex. The EDX maps of Ga and In were used to extract a line profile for both elements (left graph). The Ga signal without the DIW area was interpolated by a polynomial fit. Then, the Ga signal was normalized by dividing the Ga profile by the fit function. As a result, the right graph shows the reduction of the Ga signal within the DIW area by (12±3)%. Assuming a cylindrical shape of the DIW and the NW, the size of the DIW is about half the size of the total NW thickness at this height. Consequently, the indium content within the DIW can be roughly estimated to be (24±6)% assuming an exact stoichiometric composition.
